# Utility of hybrid PET/MRI in stereoelectroencephalography guided radiofrequency thermocoagulation in MRI negative epilepsy patients

**DOI:** 10.3389/fnins.2023.1163946

**Published:** 2023-06-12

**Authors:** Hongyang Li, Miao Zhang, Zhengyu Lin, Zhengdao Deng, Chunyan Cao, Shikun Zhan, Wei Liu, Bomin Sun

**Affiliations:** ^1^Department of Neurosurgery, Ruijin Hospital, Shanghai Jiao Tong University School of Medicine, Shanghai, China; ^2^Center for Functional Neurosurgery, Ruijin Hospital, Shanghai Jiao Tong University School of Medicine, Shanghai, China; ^3^Department of Nuclear Medicine, Ruijin Hospital, Shanghai Jiao Tong University School of Medicine, Shanghai, China; ^4^Research Group of Experimental Neurosurgery and Neuroanatomy, KU Leuven, Leuven, Belgium

**Keywords:** drug-resistant epilepsy, SEEG-guided RFTC, hybrid PET/MRI, multifocal epilepsy, epilepsy surgery

## Abstract

**Introduction:**

Hybrid positron emission tomography/magnetic resonance imaging (PET/MRI) is a novel advanced non-invasive presurgical examination tool for patients with drug-resistant epilepsy (DRE). This study aims to evaluate the utility of PET/MRI in patients with DRE who undergo stereoelectroencephalography-guided radiofrequency thermocoagulation (SEEG-guided RFTC).

**Methods:**

This retrospective study included 27 patients with DRE who underwent hybrid PET/MRI and SEEG-guided RFTC. Surgery outcome was assessed using a modified Engel classification, 2 years after RFTC. Potential areas of the seizure onset zone (SOZ) were identified on PET/MRI and confirmed by SEEG.

**Results:**

Fifteen patients (55%) became seizure-free after SEEG-guided RFTC. Engel class II, III, and IV were achieved in six, two, and four patients, respectively at the 2 years follow-up. MRI was negative in 23 patients and structural abnormalities were found in four patients. Hybrid PET/MRI contributed to the identification of new structural or metabolic lesions in 22 patients. Concordant results between PET/MRI and SEEG were found in 19 patients in the identification of SOZ. Among the patients with multifocal onset, seizure-free status was achieved in 50% (6/12).

**Conclusion:**

SEEG-guided RFTC is an effective and safe treatment for drug-resistant epilepsy. Hybrid PET/MRI serves as a useful tool for detecting the potential SOZs in MRI-negative patients and guide the implantation of SEEG electrodes. Patients with multifocal epilepsy may also benefit from this palliative treatment.

## Introduction

Stereoelectroencephalography-guided radiofrequency thermo coagulation (SEEG-guided RFTC) is a novel palliative treatment for patients with drug-resistant epilepsy (DRE). This technique was first introduced in 2004 when the SEEG electrodes were used to perform bipolar thermocoagulation of the seizure onset zone (SOZ) in patients with DRE (Guenot et al., 2004). Candidates for this treatment are usually ineligible for conventional surgical resection of the SOZ due to the proximity to eloquent areas or an extensive epileptic network (Bourdillon et al., 2018). The safety and efficacy of this technique have been acknowledged widely. A large-sampled prospective study reported a seizure-free rate of 7% and responder rate (defined as >50% decreased seizure frequency) of 48% at 1 year follow-up with permanent and transient side effect rates of 1.1 and 2.4%, respectively (Bourdillon et al., 2017). The therapeutic effects of SEEG-guided RFTC in new specific indications have increasingly been identified, including hypothalamic hamartomas and periventricular nodular heterotopia (Mirandola et al., 2017; Wei et al., 2018; Wang et al., 2020). And SEEG-guided RFTC has also been proven to be effective in treating insular epilepsy and malformation of cortical development (MCD)-related epilepsy (Catenoix et al., 2015; Mullatti et al., 2019). Compared to traditional surgery, SEEG-guided RFTC is associated with fewer surgical complications, shorter hospitalization, and better preservation of cerebral function.

Hybrid PET/MRI is an up-to-date non-invasive presurgical examination of epilepsy. It allows spatial and temporal registration of both imaging datasets, which is vital for improving MRI postprocessing and PET quantification (Pyatigorskaya et al., 2020). And it has been used in a variety of brain diseases including neurodegenerative disorders, brain tumor, and epilepsy (Garibotto et al., 2013). Extensive studies have evaluated the advantage of hybrid PET/MRI in the presurgical examination of DRE in detecting the underlying SOZs that were overlooked by standalone MRI or PET/CT (Shin et al., 2015; Oldan et al., 2018). Also, the presence of lesions on MRI could be predictive of surgical outcome after respective surgery or SEEG-guided RFTC and MRI negative patients usually achieve less satisfactory results (Immonen et al., 2010; Cossu et al., 2015; Dimova et al., 2017). However, to date few studies have investigated the use of hybrid PET/MRI in the presurgical examination of MRI negative patients with DRE who underwent SEEG-guided RFTC.

In this retrospective study, we report the experience of our center in a consecutive series of patients with DRE, who underwent hybrid PET/MRI and SEEG-guided RFTC. We hypothesized that hybrid PET/MRI could serve as a useful tool in the presurgical examination in MRI negative patients with DRE.

## Materials and methods

### Participants

This study was approved by the Ethics Committee of Ruijin Hospital, Shanghai Jiao Tong University, School of Medicine. All procedures performed in this study were in accordance with the Helsinki declaration and its later amendments. Written informed consent was obtained from all of the patients and the legal guardians of patients aged under 18. A total of 27 patients underwent an SEEG evaluation and subsequent SEEG-guided RFTC at Ruijin Hospital between August 2018 and October 2021. All patients completed a presurgical evaluation of PET/MRI and postsurgical follow-up.

### Inclusion and exclusion criteria

The inclusion criteria for the patients were as follows: (1) diagnosis of drug-resistant epilepsy based on International League Against Epilepsy (ILAE) criteria and comprehensive clinical examinations, including a comprehensive neurologic evaluation, video electroencephalogram (EEG) recordings, diagnostic MRI, PET, and SEEG; (2) underwent SEEG examination and subsequent RFTC; and (3) underwent hybrid PET/MRI examination. The exclusion criteria were as follows: (1) post traumatic epilepsy, brain tumor, or mental disorders; (2) pregnancy or breastfeeding; (3) underwent previous resection surgery, or (4) with serious head movement artifacts and difficulty in PET/MR imaging interpretation.

### PET/MRI acquisition protocol

A detailed workflow was presented in our previous research (Zhang et al., 2020). Hybrid PET/MRI scanning was performed on a scanner (Biograph mMR, Siemens Healthcare, Erlangen, Germany) using the 12-channel vendor-supplied phased-array head coil. The 3T MRI scanner was integrated into a PET ring, between the gradient coils and the shielded radio-frequency body coil. After fasting for at least 6 h, ^18^F-fludeoxyglucose (FDG) was injected into the patients. A hybrid ^18^F-FDG PET/MRI examination started 40 min after the injection. Static FDG-PET data were acquired in sinogram mode for 15 min covering the whole brain. Attenuation correction (AC) was conducted using advanced PET attenuation correction using a unique five-compartment model (Koesters et al., 2016). MRI imaging was acquired simultaneously with PET. MRI scanning was performed based on standard epilepsy MRI protocols (Wellmer et al., 2013; Urbach et al., 2015; Mouthaan et al., 2016; Deuschl et al., 2020).

### PET/MRI interpretation

Criteria of potential SOZs on MRI includes: (1) hippocampal atrophy; (2) obvious morphological alterations; (3) abnormal MRI signal on T1, T2, or Flair.

Positron emission tomography hypometabolism was identified based on standard visual analysis. All PET images were evaluated by two independent radiologists blinded to clinical information, and if they had any different opinions a third nuclear medicine physician was used to judge against discrepancies. Areas of substantial hypometabolism compared to the contralateral side were considered as potential SOZs. And brain areas with normal and symmetric distribution of metabolism were considered as negative. Concordance between PET/MRI findings and RFTC sites was defined as hypometabolic status detected in all of or part of the RFTC sites, whereas discordance was defined as no hypometabolism in RFTC locations.

### SEEG implantation and RFTC

The data obtained from non-invasive evaluations were not congruent for the localization of SOZs. Thus, SEEG implantation with recordings of spontaneous seizures was consequently undertaken. The number and placement of electrodes were defined according to the potential epileptogenic foci, which was hypothesized based on seizure semiology and the result of non-invasive investigations including VEEG, PET/MRI and after discussion with a group of experienced multidisciplinary experts.

Stereotactic electrodes placement was performed while the patient was under general anesthesia. A total of 3–12 (mean eight) multi-contact semi-rigid electrodes (Beijing Sinovation Medical Technology Co., LTD; diameter of 0.8 mm, 8–16 contacts, 2 mm length, 1.5 mm apart) were implanted per patient. A total of 25 patients received electrode implantation in bilateral hemisphere, with a right hemispheric dominance in 14 cases, a left-hemispheric dominance in seven cases, and an equal number of electrodes in each hemisphere in four cases. Two patients received electrode implantation in the right hemisphere. After implantation, the postoperative CT scan was performed to confirm the location of SEEG electrodes and ensure that there was no intracerebral hemorrhage. After the patient awaked, the intracranial recording (3–10 days) using an intensive video-EEG monitoring system (Compumedics, Abbotsford, VIC, Australia) began while the patient was on a low dose of medication. SEEG ictal onsets were considered relevant when they showed a low-voltage fast activity in the beta and gamma bands, or a recruiting and periodic fast discharge of spikes. Once monitoring was completed, recording materials were reviewed and interpreted by the epileptologists to identify the SOZ, defined as the region exhibiting the first clear SEEG change before the clinical onset of a seizure.

Indications for SEEG-guided RFTC included: (1) a SOZ that involved functionally critical areas, the resection of which could create unacceptable functional deficit; (2) the SOZ is of limited range; (3) patient refusal to undergo respective surgery; (4) detection of ictal epileptogenic discharge at seizure onset; (5) multifocal seizure onset; (6) interictal epileptiform discharge with high amplitude spikes in a repetitive fashion. The procedure was performed at the end of the recording period and before electrode withdrawal. No anesthesia was used so that the reaction of patients during the procedure could be monitored. Lesion sites where stimulation elicited movement disorders or language disorders were not coagulated. The lesioned contacts were connected to radiofrequency (RF) lesion-generator equipment (Cosman Medical Inc, Burlington, MA, USA). We adopted the following parameters: current power progressively raised from 4 to 6 W within 30 s and voltage (usually approximately 70 V) variable according to impedance. These parameters were found to increase the tissue temperature to 75^°^C. We continued SEEG monitoring for a few days after coagulation to observe changes in seizure frequencies and evaluate how interictal epileptiform activity was influenced by RFTC. The SEEG electrodes were removed at the end of the recording and the patient was discharged within 24 h. Full concordance between PET/MRI hypometabolism and SEEG findings was defined as all the RFTC sites presented with focal hypometabolism. Partial concordance was defined as only a part of the RFTC sites presented with hypometabolism while some RFTC sites do not. Discordance was defined as none of the RFTC sites presented with hypometabolism.

### Follow-up and outcome

The rate of seizure frequency reduction and the possible adverse events of the SEEG-guided RFTC were collected through clinical visits or phone calls, postoperatively. Patients’ spontaneous complaints about memory, language, attention, and movement were recorded. Seizure outcomes were classified by independent epileptologists according to Engel et al. (1993) classification.

## Results

### Patients’ demographics and clinical characteristics

Between 2018 and 2020, SEEG-guided RFTC was applied to 29 patients with DRE, among whom 27 underwent hybrid PET/MRI. The demographic data and clinical characteristics of 27 patients are shown in Table 1. The study population included 18 males and nine females. The mean age of the patient group was 25.6 ± 14.9 years (mean ± SD, range 10–70 years) and the mean age at epilepsy onset was 18.8 ± 14.7 years (mean ± SD, range 2–70 years). And the detailed patient information is presented in Table 2.

**TABLE 1:** Population characteristics.

Characteristics	Total patients
Number (N)	27
Male/female (N)	18/9
Age (years, mean ± SD)	25.6 ± 14.9
Age of seizure onset (years, mean ± SD)	18.8 ± 14.7
Duration of epilepsy (years, mean ± SD)	7.4 ± 8.8
Follow-up period (years, mean ± SD)	27.8 ± 12.7
**Seizure frequency**	
Daily	10 (37.0%)
Weekly	3 (11.1%)
Monthly	5 (18.5%)
Yearly	9 (33.3%)
**MRI performance**	
Normal	23
Gray matter heterotopia	1
Hippocampal atrophy	2
Polymicrogyria	1
**PET/MRI performance**	
Normal	3
Abnormal	24
**Site of SOZs**	
Temporal	17
Frontal	11
Parietal	3
Occipital	5
Insular	2
Focal seizure onset	15
Multifocal seizure onset	12
Number of implanted SEEG electrodes (mean ± SD)	8.9 ± 2.4

**TABLE 2:** Detailed clinical profiles in 27 patients with epilepsy.

Case	Age/sex	Epilepsy duration, year	Follow-up, month	Engel class	MRI finding	MRI portion of hybrid PET/MRI	PET portion of hybrid PET/MRI	Reasons for RFTC	RFTC locations	Concordance between PET/MRI and RFTC
1	26/M	6	9	I	Gray matter heterotopia in R. frontal lobe	Gray matter heterotopia in R. middle frontal gyrus; R. hippocampal atrophy	Increased FDG in R. middle frontal gyrus; decreased FDG in B. temporal lobe;	Multifocal onset; IED	R. frontal lobe, R. temporal lobe,	Partial concordant
2	16/F	5	10	I	Neg	Neg	Decreased FDG in B. temporal lobe	Multifocal onset; eloquent cortex;	R. precentral gyrus, R. superior and middle frontal gyrus and anterior insula	Discordant
3	20/M	6	24	I	Neg	Neg	Decreased FDG in B. temporal lobe	Multifocal onset; eloquent cortex; IED	R. occipital lobe and precentral gyrus	Discordant
4	30/F	9	24	I	Neg	R. hippocampal atrophy	Decreased FDG in B. temporal lobe;	Refusal to respective surgery; IED	R. amygdala and hippocampus	Fully concordant
5	13/M	2	24	I	Neg	R. hippocampal atrophy	Neg	Refusal to respective surgery; IED	L. frontal lobe	Discordant
6	17/M	15	34	I	Neg	R. hippocampal atrophy	Increased FDG in B. frontal lobe and decreased FDG in B. temporal and occipital lobe	Eloquent cortex; IED	R. occipital lobe	Fully concordant
7	36/F	20	44	I	Neg	Neg	Decreased FDG in L. cingulate, B. temporal and frontal lobe	Multifocal onset; eloquent cortex; IED	L. occipital lobe and L. temporal lobe	Partial concordant
8	10/M	3	47	I	Neg	Neg	Decreased FDG in L. supramarginal gyrus	Refusal to respective surgery; IED	L. frontal lobe	Fully concordant
9	18/F	3	47	I	Neg	R hippocampal atrophy	Decreased FDG in L. temporal lobe and B. frontal lobe;	Eloquent cortex; IED	R. postcentral gyrus	Discordant
10	32/M	2	34	I	Neg	Neg	Decreased FDG in B. frontal and parietal lobe	Multifocal onset; eloquent cortex; IED	L. hippocampus and amygdala; R. postcentral gyrus	Partial concordant
11	21/F	2	30	I	Neg	Neg	Decreased FDG in R. temporal lobe	Refusal to respective surgery	R. temporal lobe	Fully concordant
12	30/M	8	44	I	B. hippocampal atrophy	Neg	Decreased FDG in R. temporal and B. frontal lobe	Eloquent cortex; IED	L. precentral gyrus	Fully concordant
13	13/M	12	48	I	Neg	Neg	Neg	Refusal to respective surgery; IED	L. parietal lobe	Discordant
14	19/M	2	27	I	Neg	Bilateral hippocampal atrophy	Decreased FDG in L. inferior temporal gyrus	Eloquent cortex	L. hippocampus and temporal lobe	Fully concordant
15	19/M	4	7	I	Neg	R. hippocampal atrophy	Decreased FDG in R. temporal lobe	Multifocal onset	R. hippocampus and amygdala; R. occipital lobe	Partial concordant
16	10/M	2	25	II	Neg	Neg	Decreased FDG in R. precentral gyrus	Refusal to respective surgery; IED	R. cingulate	Discordant
17	26/M	10	15	II	Neg	Enlarged R. anterior temporal lobe	Decreased FDG in R. temporal lobe	Multifocal onset; IED	R. frontal and temporal lobe	Partial concordant
18	15/F	11	10	II	Polymicrogyria in B. inferior frontal gyrus	Polymicrogyria in B. inferior frontal gyrus	Decreased FDG in B. inferior frontal gyrus and B. temporal lobe	Multifocal onset	B. inferior frontal gyrus	Fully concordant
19	25/F	2	28	II	Neg	Neg	Decreased FDG in B. frontal and temporal lobe	Multifocal onset; eloquent cortex; refusal to respective surgery	B. temporal lobe	Fully concordant
20	30/F	3	23	II	Neg	L. hippocampal atrophy	Decreased FDG in L. temporal lobe	Eloquent cortex	L. temporal lobe	Fully concordant
21	32/M	2	31	II	Neg	Neg	Decreased FDG in R. frontal and temporal lobe	Refusal to respective surgery	R. temporal lobe	Fully concordant
22	54/M	2	12	III	Neg	Atrophy in B. temporal and parietal lobe	Decreased FDG in L. temporal lobe;	Refusal to respective surgery	L. temporal lobe	Fully concordant
23	59/F	44	25	III	Neg	L. hippocampal atrophy; Atrophy in B. temporal lobe	Decreased FDG in L. temporal lobe	Eloquent cortex; IED	L. temporal lobe	Fully concordant
24	16/M	7	15	IV	R. hippocampal atrophy	R. hippocampal atrophy	Decreased FDG in R. temporal lobe;	Multifocal onset; IED	R. frontal and temporal lobe;	Partial concordant
25	31/M	15	31	IV	Neg	B. hippocampal atrophy;	Decreased FDG in B. temporal lobe	Multifocal onset; eloquent cortex	R. frontal and temporal lobe	Partial concordant
26	10/M	2	38	IV	Neg	Neg	Neg	Multifocal onset	R. temporal and frontal lobe;	Discordant
27	70/M	2	45	IV	Neg	Neg	Increased FDG in R. hippocampus and amygdala	Eloquent cortex	L. temporal lobe	Discordant

B, bilateral; L, left; R, right; IED, interictal epileptiform discharge indicative of seizure onset zone (SOZ).

### Seizure outcome and complications

The mean SEEG electrode number in the patients was 9.1 ± 2.3 (mean ± SD, range 3–12). The mean follow-up period was 27.8 ± 12.7 (mean ± SD, range 7–47) months. The mean duration of epilepsy was 7.4 ± 8.8 (mean ± SD, range 2–44) years. SEEG-guided RFTC was conducted in all patients as a palliative treatment to reduce the frequency of seizures and their impact on daily life. A total of 15 patients became seizure-free after RFTC and were classified as Engel class I. Engel class II and III were achieved in six and two patients, respectively. Four patients who did not show any improvement were classified as Engel class IV. The seizure free rate was 55% at a mean follow-up period of 21 months. No permanent sequelae were observed in any patients. Three patients who underwent RFTC in the left hippocampus complained of transient verbal memory deficit. One patient, in whom RFTC was performed in the left central region, presented with a transient motor deficit in her right arm, which resolved spontaneously within a month.

### PET/MRI and SEEG results

**FIGURE 1:**
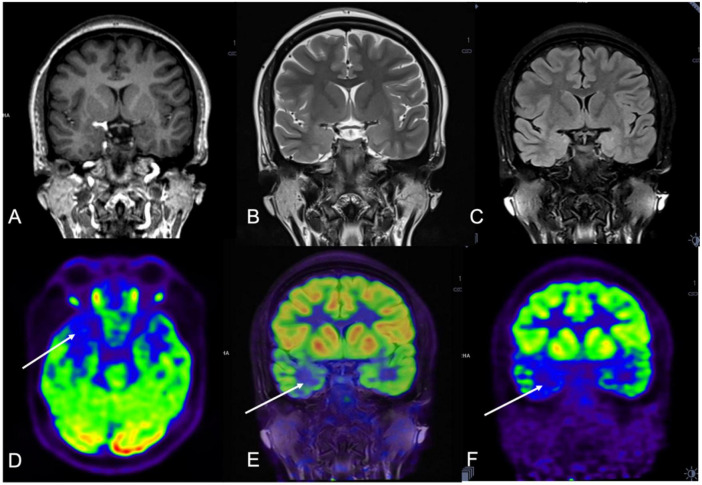
A 21-year-old woman (patient #11) with refractory epilepsy for 1 year. Magnetic resonance (MR) imaging coronal T1 **(A)**, coronal T2 **(B)**, and coronal Flair **(C)** were negative. Positron emission tomography (PET) **(D,F)** and hybrid PET/MRI **(E)** showed focal hypometabolic area in the right temporal lobe. Combined with semiology and stereoelectroencephalography (SEEG) results, the right mesial temporal lobe was hypothesized to be the seizure onset zone (SOZ) and stereoelectroencephalography-guided radiofrequency thermo coagulation (SEEG-guided RFTC) was performed. And the patient was seizure-free at 30-month follow-up.

**FIGURE 2:**
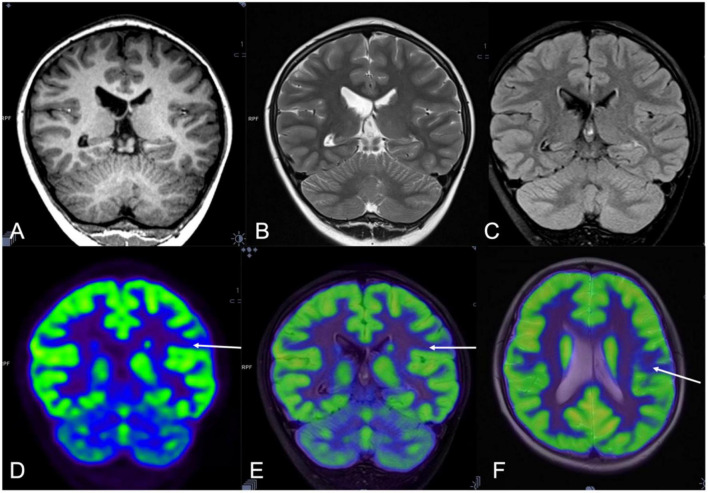
A 10-year-old man (patient #8) with refractory epilepsy for 3 years. Magnetic resonance (MR) imaging coronal T1 **(A)**, T2 sequence **(B)**, and Flair images **(C)** were negative. Positron emission tomography (PET) **(D)** and hybrid PET/MRI showed focal hypometabolic area in the left supramarginal gyrus **(E,F)**. Combined with semiology and stereoelectroencephalography (SEEG) results, the left supramarginal gyrus was supposed as the seizure onset zone (SOZ) and stereoelectroencephalography-guided radiofrequency thermo coagulation (SEEG-guided RFTC) was performed. And the patient was seizure-free at the 47-month follow-up.

magnetic resonance imaging was negative in 23 patients and structural abnormalities were found in four patients, including gray matter heterotopia and polymicrogyria in one patient each and hippocampal atrophy in two patients. Abnormal FDG uptake was found in 24 patients and a normal PET result was reported in three patients. Hybrid PET/MRI contributed to the identification of new structural or metabolic lesions in 22 patients (See Figures 1, 2). The new structural lesions on MRI were seen in 12 patients which concorded with the PET findings in eight (See Table 2). The PET/MRI images were imported into Leksell surgiplan software to navigate the implantation of SEEG electrodes and PET/MRI guided the implantation of SEEG electrodes in all the patients who presented with abnormal PET/MRI.

**FIGURE 3:**
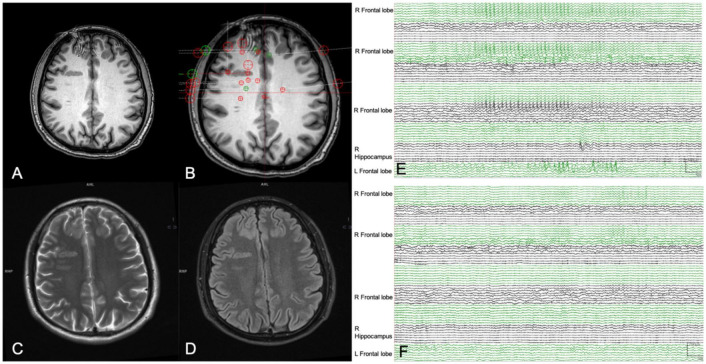
A 26-year-old man (patient #1) with refractory epilepsy for 6 years. Magnetic resonance (MR) imaging axial T1 **(A)**, axial T2 **(C)**, and axial Flair **(D)** indicated grey matter heterotopia in the right frontal lobe. Stereoelectroencephalography (SEEG) electrodes were implanted to sample all the suspicious seizure onset zone (SOZs) **(B)**. Yet a habitual seizure attack failed to be captured by SEEG. Presurgical interictal SEEG recording **(E)** showed high-frequency spikes in bilateral frontal lobe and right hippocampus. Combined with semiology and MRI images, the bilateral frontal lobe the right hippocampus was hypothesized to be the suspicious SOZ and stereoelectroencephalography-guided radiofrequency thermo coagulation (SEEG-guided RFTC) was performed. Epileptiform discharges disappeared after RFTC during SEEG monitoring **(F)**. And the patient was seizure-free at 9-month follow-up.

During the SEEG recording period, a habitual seizure attack failed to be captured in 15 patients. These patients had low preoperative seizure frequency, with 10 patients only had several seizure attacks per year. Interictal SEEG recording reported spikes of high amplitude in a repetitive fashion in these patients. With the support of other information including PET/MRI and semiology, brain areas highly suspicious of SOZ were identified (See Figure 3). In the other 12 patients, at least one habitual seizure attack was captured during SEEG recording. The SOZ was found in temporal lobe in 17, frontal lobe in 11, parietal lobe in three, occipital lobe in five and insular in two patients. To sum up, multifocal SOZs was found in 12 patients while focal SOZs was found in 15 patients.

Stereoelectroencephalography-guided RFTC was applied in 13 patients with an SOZ overlapping with eloquent cortex as a palliative technique. SOZ locations were fully concordant between PET/MRI and SEEG in 12 patients. Partial concordance was found in seven patients, which means PET/MRI only found a part of SOZs while other SOZs remain undetected by PET/MRI. Discordant findings were found in eight patients, which means PET/MRI failed to detect the underlying SOZs. To sum up, fully and partial concordance between the two modalities were found in 17 among the 27 patients. As the SEEG examination is the gold standard in defining the SOZs in patients with epilepsy, the sensitivity of PET/MRI in localizing the SOZs was 70% in our cohort. In patients with focal onset, seizure free was achieved in 60% (9/15) and Engel II, III, and IV were achieved in 3, 2, and 1 patient, respectively. And among the patients with multifocal onset, seizure free was achieved in 50% (6/12) at 2 years follow-up and Engel II, III, and IV were achieved in 3, 0, and 3 patients, respectively. And among the patients with only interictal epileptiform discharge indicative of SOZs, seizure freedom was achieved in 80% (12/15) at 2 years follow-up.

## Discussion

Fifteen patients (55%) became seizure-free after RFTC procedure and Engel class II, III, and IV were achieved in six, two, and four patients, respectively, at the 2 years follow-up. The patients included in this case series have relatively short history of epilepsy and low seizure frequency preoperatively, which may account for the high rate of seizure-free rate at 2 years follow-up compared to previous studies (Bourdillon et al., 2017; Chipaux et al., 2019). MRI was negative in 23 patients and structural abnormalities were found in four patients. Hybrid PET/MRI contributed to the identification of new structural or metabolic lesions in 22 patients. Concordant results between PET/MRI and SEEG was found in 19 patients in the identification of SOZ. Among the patients with multifocal onset, seizure freedom was achieved in 50% (6/12).

Hybrid PET/MRI is a state-of-art technology that allows the acquisition of morphological and metabolic information simultaneously. And the advantage of hybrid PET/MRI in the presurgical examination of drug resistant epilepsy has been acknowledged. It yields increased diagnostic rate of SOZs compared to standalone MRI or PET/CT and provide additional sensitivity, accuracy and better image quality (Shin et al., 2015; Oldan et al., 2018; Kikuchi et al., 2021). Besides, the value of hybrid PET/MRI in predicting the outcome of epilepsy surgery has also been recognized. In a study by Guo et al. (2021) concordant results between surgical resection range and hypometabolic area on hybrid PET/MRI could predict seizure freedom (odds ratio = 14.741; 95% CI = 3.934–55.033, *p* < 0.001). The sensitivity, specificity, and accuracy of PET/MRI in identifying the SOZs were reported to be 95.3, 8.8, and 65.3%, respectively. In our patient cohort, 23 of 27 patients were non-lesional on standalone MRI. Hybrid PET/MRI contributed to the identification of new structural or metabolic lesions in 22 patients. New structural lesions on MRI were seen in 12 patients. And the new structural lesions were concordant with PET findings in 8 patients. Hybrid PET/MRI navigated the implantation of SEEG electrodes in 25 patients and revealed the same SOZ location in 19 patients.

In clinical practice, PET could serve as a complementary presurgical examination to EEG for SOZ identification. Johnson et al. (2020) revealed that concordance of SOZ localization between PET and EEG was associated with favorable surgical outcomes in almost all patients while discordance had equal chance of favorable or unfavorable outcomes. According to Desarnaud et al. (2018) the localization rate of SOZs was 83% after integration of electroclinical data and PET/MRI in patients with pathologically proven focal cortical dysplasia type 2 (FCD2), significantly higher than that of PET or EEG alone. According to a study by our group, concordant results between SEEG and PET/MRI was associated with satisfactory outcome (odds ratio = 20.41; 95% CI = 2.75–151.4; *p* = 0.003) (Zhang et al., 2020). In our patient cohort, concordant results between PET/MRI and SEEG was found in 19 patients and the sensitivity of PET/MRI in localizing the SOZ was 70%. Among the 19 patients with concordant findings between the two modalities, seizure freedom was achieved in eight (42%). PET/MRI is better in expanding the range of possible SOZs rather than confirming a specific EZ to be resected based on that the hypometabolism area usually extend to a larger area beyond the structural lesion (Theodore et al., 1986; Ryvlin et al., 1992). SEEG findings could be used when presurgical examination were not congruent and hybrid PET/MRI alone cannot serve as a definite prognostic factor in epileptic patients.

Owing to the paroxysmal nature of seizure onset, a habitual seizure attack may fail to be captured by EEG or SEEG, especially in those with low preoperative seizure frequency. In this case, interictal epileptiform discharge could be used to support the diagnosis of epilepsy and the identification of SOZ. And it is pointed out that spikes of high amplitude and repetitive fashion are often related to an epileptogenic process (Lüders, 2008). Combined with other information including semiology and PET/MRI, we could identify the brain areas that are highly suspicious of seizure onset. Among the 15 patients with RFTC in the “suspicious SOZ,” 12 achieved Engel I at 2 years follow-up.

Multifocal epilepsy has been a challenge in the clinical practice of epilepsy surgery. The presence of multifocal epilepsy ranks as the second most common reason for not having epilepsy surgery, accounting for 24% of all patients, preceded by inability to localize the SOZs (Khoo et al., 2021). Respective surgery has been attempted in patients with multifocal epilepsy involving eloquent cortex. However, unsatisfying outcome was obtained with Engel class I only achieved in 31% (4/13) at a mean of 59 months follow-up (Maksymowicz et al., 2005). Recently neurostimulation therapies, including deep brain stimulation (DBS), responsive neurostimulation (RNS), and vagal nerve stimulation (VNS) have been used in the treatment of multifocal epilepsy (Giordano et al., 2017; Sisterson et al., 2019; Rincon et al., 2021). Although a promising outcome was obtained with seizure frequency reduction of >50% in most of the included patients, the sample in these studies was exceedingly small, thus limiting the generalizability of the efficacy of neuromodulation for patients with multifocal epilepsy (Elder et al., 2019; Theroux et al., 2020; Herrera et al., 2021). The implantation of DBS or VNS devices could bring about risks of complications including infection and rejection. And postoperative programming could be complex and time-consuming. SEEG-guided RFTC is a safe procedure and has also been tried as a palliative therapy in the treatment of patients with multifocal epilepsy (Cossu et al., 2015; Dimova et al., 2017; Mereaux et al., 2020). In our cohort, seizure freedom was achieved in 50% (6/12) at 2 years follow-up among the patients with multifocal onset after SEEG-guided RFTC. We propose that SEEG-guided RFTC at several suspicious SOZs could interfere with complex epileptogenic networks and hindered the initiation and spread of ictal discharge. And SEEG-guided RFTC is more affordable and acceptable compared with neuromodulation and could serve as an alternative treatment for patients with multifocal epilepsy.

There are some disadvantages associated with SEEG-guided RFTC. First, only limited lesion volume was produced by RFTC. We adopted a lesioning parameter of heating temperature at 80^°^C and maintain this level for 30 s. And it is suggested that different RFTC parameters, for example, duration time of RFTC procedure, number of RFTC lesioning contacts and speed of power increase may have an impact on the lesion volume (Chipaux et al., 2019). Second, SEEG electrodes are implanted in confined areas based on presurgical hypotheses of the possible localization of SOZs. Thus, some SOZs may be overlooked.

This study has some limitations. First, the small sample size and relatively short follow-up duration. Second, this a single-center retrospective study. Finally, the patients included in the study had a relatively short history of epilepsy and low seizure frequency preoperatively which may account for a higher the seizure-free rate at 2 years follow-up than other previous studies.

## Conclusion

The SEEG-guided RFTC is an effective and safe treatment for drug-resistant epilepsy. Hybrid PET/MRI serves as a useful tool for detecting the potential SOZs in MRI-negative patients and guide the implantation of SEEG electrodes. Patients with multifocal epilepsy may also benefit from this palliative treatment.

## Data availability statement

The original contributions presented in this study are included in this article/supplementary material, further inquiries can be directed to the corresponding authors.

## Ethics statement

The studies involving human participants were reviewed and approved by Ethics Committee of Ruijin Hospital, Shanghai Jiao Tong University, School of Medicine. Written informed consent to participate in this study was provided by the participants’ legal guardian/next of kin.

## Author contributions

HL and ZL wrote the manuscript. ZD was responsible for the follow-up of the patients. MZ contributed to the acquisition and interpretation of PET/MRI imaging. SZ, BS, and WL performed the neurosurgery. CC took part in the presurgical evaluation of epilepsy patients. All authors contributed to the article and approved the submitted version.
